# Supplementary material for: Transcriptome Profiling of Oncorhynchus mykiss Infected with Low or Highly Pathogenic Viral Hemorrhagic Septicemia Virus (VHSV)
Source: Microorganisms. 2023 Dec 28;12(1):57. doi: 10.3390/microorganisms12010057 (PMC10821180; doi:10.3390/microorganisms12010057)
Supplement: Supplementary file 1 [file microorganisms-12-00057-s001.zip › Supplementary Table_S6.pdf]

Table S6: Top Up and Down-regulated DEGs at each time point for each of the VHSV-H and VHSV-L groups. 1

VHSV-H 1dpi

Differential Gene Expression Trend	Gene name	Gene description	Database identifier Number			log2FC
			ZFIN: ZDB-GENE-Id	Uniprot	NCBI: Gene ID	
Up-regulated	ctrb.3	chymotrypsinogen B, tandem duplicate 3	050417-229	Q561U4 P17538	110490652	35,39
	defb3	Oncorhynchus mykiss defensin beta 3	070207-3	C9WX70 P81534	100499616	34,33
	ela3l	elastase 3 like	060710-2	A0A8M9QDY3 P09093		30,73
	ins	Oncorhynchus mykiss preproinsulin 1	980526-110	A3RID6	100136702	28,08
	/	trypsinogen-like protein 3			110537956	26,62
	uox	uricase-like	030826-24	Q6DG85	110521955	26,18
	C1QL1	C1q-related factor-like		O75973	110491703	23,20
	APOB	apolipoprotein B-100-like		P04114	110529382	22,82
	ins	preproinsulin	980526-110	O73727 P01308	30262	22,00
	trim63a	tripartite motif containing 63a	040625-139	Q6IQH8 Q969Q1		21,29
Down-regulated	gck	glucokinase (hexokinase 4)	060825-204	P35557		-40,22
	hbbe2	Oncorhynchus mykiss embryonic beta-type globin2		Q9YGF4	100135792	-30,69
	degs2-like	sphingolipid delta(4)-desaturase/C4-monooxygenase DES2-like			110533418	-25,74
	/	late histone H2A.2.2-like	050522-93		110495475	-25,57
	b3galt11	beta-1,3-galactosyltransferase 1-like	030131-4878	B3DK04 Q9Y5Z6	110485553	-24,98
	rad21l1	double-strand-break repair protein rad21 homolog	061103-505	O60216	110492763	-24,88
	tspan13a	tetraspanin-13-like	040718-19	Q6DG37 O95857	110516043	-24,47
	vtg3	vitellogenin-like	991019-2		110521946	-23,89
	sox19b	transcription factor Sox-19b-like	010111-1	Q9DDD7	110500375	-23,85
	fam149a	family with sequence similarity 149 member A	081107-36		100001989	-23,72

VHSV-H 2dpi

Differential Gene Expression Trend	Gene name	Gene description	Database identifier Number			log2FC
			ZFIN: ZDB-GENE-Id	Uniprot	NCBI: Gene ID	
Up-regulated	fam32a	family with sequence similarity 32 member A	040704-44	Q9Y421	431750	36,75
	/	ladderlectin-like		E7FAQ3<br>P81018	110527149	32,30
	/	trypsinogen-like protein 3			110537956	31,05
	hbbe2	Oncorhynchus mykiss embryonic beta-type globin2		Q9YGF4	100135792	24,95
	ins	Oncorhynchus mykiss preproinsulin 1	980526-110	A3RID6	100136702	24,00
	/	si:ch211-240l19.6	041210-328			23,64
	tdrd7a	tudor domain containing 7 a	040426-2103	A6NAF9<br>Q8NHU6	406379	21,93
	lhx9	LIM/homeobox protein Lhx9-like	050417-210	Q1LWV4<br>Q9NQ69	110524779	20,72
	gata4	GATA binding protein 4	980526-476		110522338	19,76
	/	phospholipase A2, minor isoenzyme-like			110525547	19,60
Down-regulated	uox	uricase-like	030826-24	Q6DG85	110521955	-27,02
	/	GTPase IMAP family member 4-like		Q8NHV1	110521971	-26,83
	vtg3	vitellogenin-like	991019-2		110521946	-24,76
	APOB	apolipoprotein B-100-like		P04114	110529382	-24,12
	tgm2l	protein-glutamine gamma-glutamyltransferase 2-like	050420-97	P21980	110520123	-19,21
	sdc4	syndecan-4-like	061111-1	P31431	110493836	-17,47
	LYPD6	ly6/PLAUR domain-containing protein 6-like	040912-117	Q86Y78	110495391	-16,13
	MTNR1A	melatonin receptor Mel1a	990415-155	P51046<br>P48039	100135888	-16,10
	/	smoothelin-like protein 2		Q2TAL5	110537678	-15,80
	epyc	epiphycan	041008-9	Q99645		-15,66

VHSV-H 5dpi

Differential Gene Expression Trend	Gene name	Gene description	Database identifier Number			log2FC
			ZFIN: ZDB-GENE-Id	Uniprot	NCBI: Gene ID	
Up-regulated	apoda.2	apolipoprotein D-like	070912-551		110509046	39,06
	hbbe2	Oncorhynchus mykiss embryonic beta-type globin2		Q9YGF4	100135792	28,27
	degs2-like	sphingolipid delta(4)-desaturase/C4-monooxygenase DES2-like		Q6QHC5	110533418	24,86
	tdrd7a	tudor domain containing 7	040426-2103	A6NAF9<br>Q8NHU6	406379	24,81
	nmbr	neuromedin B receptor	041014-369	P28336		23,84
	tdrd1	tudor domain containing 1	070803-1	Q58EK5	553522	23,47
	lhx8a	LIM/homeobox protein Lhx8-like	031008-2	Q6BDC3	110530197	23,20
	vtg3	vitellogenin-like	991019-2		110521946	23,14
	b3galt1l	beta-1,3-galactosyltransferase 1-like	030131-4878	B3DK04<br>Q9Y5Z6	110485553	23,04
	tspan13a	tetraspanin-13-like	040718-1		110516043	22,27
Down-regulated	vtnb	vitronectin b	041116-1	P04004	100136138	-41,48
	gck	glucokinase (hexokinase 4)	060825-204	P35557		-38,82
	ela3l	elastase 3 like	060710-2	P09093		-32,00
	uox	uricase-like	030826-24	Q6DG85	110521955	-26,79
	ttc14	tetratricopeptide repeat domain 14	050327-26	Q96N46		-26,12
	/	low choriolytic enzyme-like			110526218	-18,99
	/	cysteine-rich venom protein ophanin-like			110487915	-18,24
	DCC	DCC netrin 1 receptor	011101-2	P43146	569360	-18,22
	rhbg2a	Oncorhynchus mykiss ammonium transporter Rh type B		Q7T070<br>Q9H310	100191034	-17,06
	fabp1a	fatty acid-binding protein, liver	020318-3	P07148	110488072	-16,99

VHSV-L 1dpi

Differential Gene Expression Trend	Gene name	Gene description	Database identifier Number			log2FC
			ZFIN: ZDB-GENE-Id	Uniprot	NCBI: Gene ID	
Up-regulated	ctrb.3	chymotrypsin B-like	050417-229	Q561U4<br>P17538	110490652	40,97
	ela3l	elastase 3 like	060710-2	A0A8M9QDY3<br>P09093		37,42
	defb3	Oncorhynchus mykiss defensin beta 3	070207-3	C9WX70<br>P81534	100499616	35,50
	/	trypsinogen-like protein 3			110537956	32,52
	uox	uricase-like	030826-24	Q6DG85	110521955	26,71
	APOB	apolipoprotein B-100-like	P04114	110529382		23,98
	C1QL1	C1q-related factor-like		O75973	110491703	22,23
	ins	preproinsulin	980526-110	A3RID6	30262	20,38
	tgm2l	protein-glutamine gamma-glutamyltransferase 2-like	050420-97	P21980	110520123	20,13
	/	fucolectin-6-like	131127-215		110514348	18,68
Down-regulated	sdc4	syndecan-4-like	061111-1	Q0JY12	110493836	-23,95
	dlx2a	homeobox protein Dlx2a-like	980526-212	P50574<br>Q07687	110501453	-18,73
	arl4d	ADP-ribosylation factor-like 4D	030131-2293	P49703		-17,70
	prom2	prominin-2-like	080723-16		110536260	-17,33
	/	serine protease 27-like		Q9BQR3	110522136	-17,15
	epyc	epiphycan	041008-9	Q99645		-16,99
	snai2	zinc finger protein SNAI2-like	030326-6	O43623	110526293	-16,98
	zgc:110045	poly(rC)-binding protein 3-like			110526533	-16,96
	klhl41a	kelch-like family member 41a	081105-22	E9QIN8<br>O60662	100148315	-16,59
	MTNR1A	melatonin receptor Mel1a	990415-155	P51046<br>P48039	100135888	-16,57

VHSV-L 2dpi

Differential Gene Expression Trend	Gene name	Gene description	Database identifier Number			log2FC
			ZFIN: ZDB-GENE-Id	Uniprot	NCBI: Gene ID	
Up-regulated	fam32a	family with sequence similarity 32 member A	040704-44	Q9Y421	431750	37,49
	/	ladderlectin-like		E7FAQ3 P81018	110527149	34,40
	avd	avidin	140205-1	A0A8M1P1N5		21,17
	/	5-hydroxytryptamine receptor 1F-like		P30939	110520361	18,78
	/	L-rhamnose-binding lectin CSL3-like			110528627	18,74
	slc4a3	anion exchange protein 3-like	141006-1	A0A096X8J7 P48751	110501380	18,21
	foxa1	hepatocyte nuclear factor 3-alpha-like	990415-78	P55317	110505788	17,75
	shank2b	SH3 and multiple ankyrin repeat domains 2b	061207-58	A2AV32		17,75
	tdrd7a	tudor domain containing 7 a	040426-2103	A6NAF9	406379	17,21
	gnrhr4	gonadotropin-releasing hormone II receptor-like			110520912	17,11
Down-regulated	defb3	Oncorhynchus mykiss defensin beta 3	070207-3	C9WX70 P81534	100499616	-36,52
	zgc:112160	chymotrypsin B-like	030131-1171		110490652	-34,35
	/	trypsin-like			110510128	-33,97
	ela3l	elastase 3 like	060710-2	A0A8M9QDY3 P09093		-30,85
	uox	uricase-like	030826-24	Q6DG85	110521955	-27,11
	/	GTPase IMAP family member 4-like		Q9NUV9	110521971	-26,85
	/	myosin heavy chain, fast skeletal muscle-like			110494114	-22,41
	C1QL1	C1q-related factor-like		O75973	110491703	-22,17
	fhl5	four and a half LIM domains protein 2-like	040822-3		110506255	-20,67
	klhl41a	kelch-like protein 41a	081105-22	E9QIN8 O60662	100148315	-19,89

VHSV-L 5dpi

Differential Gene Expression Trend	Gene name	Gene description	Database identifier Number			log2FC
			ZFIN: ZDB-GENE-Id	Uniprot	NCBI: Gene ID	
Up-regulated	apoda.2	apolipoprotein D-like	070912-551		110509046	40,98
	hbbe2	Oncorhynchus mykiss embryonic beta-type globin2		Q9YGF4	100135792	30,12
	nmbr	neuromedin B receptor	041014-369	A0A8N7UR00<br>P28336		26,27
	degs2-like	sphingolipid delta(4)-desaturase/C4-monooxygenase DES2-like		Q6QHC5	110533418	25,94
	tdrd7a	tudor domain containing 7 a	040426-2103	A6NAF9	406379	25,74
	APOB	apolipoprotein B-100-like		P04114	110529382	24,88
	tdrd1	tudor domain containing 1	070803-1	Q58EK5	553522	24,87
	lhx8a	LIM/homeobox protein Lhx8-like	031008-2	Q6BDC3	110530197	24,05
	tspan13a	tetraspanin-13-like	040718-19	Q6DG37<br>O95857	110516043	23,59
	b3galt11	beta-1,3-galactosyltransferase 1-like	030131-4878	B3DK04<br>Q9Y5Z6	110485553	23,53
Down-regulated	apoa1	apolipoprotein A-I-like		O42363<br>P02647	110520383	-28,25
	uox	uricase-like	030826-24	Q6DG85	110521955	-27,43
	/	cysteine-rich venom protein ophanin-like			110487915	-18,26
	klhl41a	kelch-like protein 41a	081105-22	E9QIN8<br>O60662	110520088	-17,81
	rhcg	Oncorhynchus mykiss Rhesus blood group, C glycoprotein	070424-81	Q8JI14<br>Q9UBD6	100136259	-16,56
	tnni2b.1	troponin I, fast skeletal muscle-like	050417-49		110526835	-16,42
	rnf207b	ring finger protein 207b	080227-9	E9QHE3	568322	-15,98
	gcm2	chorion-specific transcription factor GCMb-like	050127-1	Q5TLZ9<br>O7560	110535247	-15,58
	NKX2-3	homeobox protein Nkx-2.3-like	990415-178	Q8TAU0	110518739	-15,26
	MTNR1A	melatonin receptor Mel1a	990415-155	P51046<br>P48039	100135888	-15,24
