# Supplementary material for: Transcriptome Profiling of Oncorhynchus mykiss Infected with Low or Highly Pathogenic Viral Hemorrhagic Septicemia Virus (VHSV)
Source: Microorganisms. 2023 Dec 28;12(1):57. doi: 10.3390/microorganisms12010057 (PMC10821180; doi:10.3390/microorganisms12010057)
Supplement: Supplementary file 1 [file microorganisms-12-00057-s001.zip › Supplementary_Tables_S1_S2.pdf]

Supplementary Table S1: Oligonucleotides used in the present study. Nucleotide sequences, position with respect to the reference sequence, application and amplicon size are reported. The underlined sequence refers to the T7 promoter sequence used for RNA *in vitro* transcription.

Primer	Sequence 5'→ 3'	Position[a]	Application	Amplicon size (bp)	Reference
VHS_RNA_Sint_For_T7	TAATACGACTCACTATAGGGCTTGATGACATGCTCCCTTCTG	393-414	RT-PCR	470 bp	This study
VHS_RNA_Sint_Rev	CTCATCATGATGGCGTAGCG	823-842			This study
FW	AAACTCGCAGGATGTGTGCGTCC	532-554	rRT-PCR<br>qRT-PCR	77 bp	Jonstrup et al., 2013
BW	TCTGCGATCTCAGTCAGGATGAA	586-608			Jonstrup et al., 2013
Probe	6FAM-TAGAGGGCCTTGGTGATCTTCTG-BHQ-1	559-581			Jonstrup et al., 2013

[a] Nucleotide positions are in accordance with the VHSV sequence of strain DK-F1 under the GenBank accession number MT162452

Supplementary Table S2: Reaction conditions of molecular amplification viral assay applied in this study.

Method	Application	Reaction mixture				Thermal profile			
		Commercial Kit name	Reagent PCR	Final concentration	Final volume reaction (µl)	Step number	Temperature (°C)	Time	Number of Cycles
**rRT-PCR**	**Qualitative VHSV assay**	QuantiTect Probe RT-PCR	2x RT-PCR master mix	1x	25	1	50	30’	1
			QuantiTect RT Mix	1 U/reaction		2	95	15’	1
			Primer FW	900 nM		3	94	15”	40
			Primer REV	900 nM			* 60	40”	
			Probe	250 nM			72	20”	
**qRT-PCR**	**Quantitative VHSV assay**	QuantiTect Multiplex RT-PCR	2x RT-PCR master mix	1x	25	1	50	20’	1
			QuantiTect Multiplex RT Mix	0.2 µl/reaction		2	95	15’	1
			Primer FW	600 nM		3	94	45”	45
			Primer REV	600 nM			* 60	45”	
			Probe	250 nM					
**RT-PCR**	**Template creation for standard RNA *in vitro* synthesis**	OneStep RT-PCR Kit	5x Buffer	1x	25	1	50	30’	1
			OneStep RT-PCR	0.5 µl/reaction		2	95	15’	1
			RNase Inhibitor	8 U/reaction		3	94	30”	40
			dNTPs mix	400 µM			57	1’	
			VHS_RNA_Sint_For_T7	600 nM			72	1’	
			VHS_RNA_Sint_Rev	600 nM		4	72	10’	1

*Fluorescence acquisition step
